# Psychological Intervention Strategies in Adolescents with Asthma: A Review of the Literature

**DOI:** 10.3390/children13020181

**Published:** 2026-01-28

**Authors:** Esther Rodríguez-Jiménez, Javier Martín-Ávila, Selene Valero-Moreno, Marián Pérez-Marín

**Affiliations:** Department of Personality, Assessment and Psychological Treatments, Faculty of Psychology and Speech Therapy, University of Valencia, 46010 Valencia, Spain

**Keywords:** asthma, adolescence, psychological interventions, chronic illness, quality of life

## Abstract

**Highlights:**

**What are the main findings?**

- Psychological interventions for adolescents with asthma are limited, with most programs focusing on treatment adherence and educational aspects, while essential components such as emotional regulation, cognitive coping, and self-efficacy—which are critical given the bidirectional relationship between physical and psychological well-being and their impact on medical outcomes—are often neglected.
- Few studies incorporate robust theoretical frameworks or consider asthma comorbidities, highlighting substantial gaps in intervention design and scope.

**What are the implications of the main findings?**

- There is a need for evidence-based, theoretically grounded interventions that target psychological processes critical for adherence, coping, and quality of life, supporting more comprehensive clinical care for adolescents with asthma.
- Future programs should consider the heterogeneity of asthma severity and its comorbidities, as well as quality-of-life indicators, to promote positive psychosocial adjustment and enhance the overall effectiveness of medical treatment.

**Abstract:**

**Background/Objectives**: Asthma is one of the most prevalent chronic diseases in adolescence, a stage in which its management may be affected by developmental changes. The aim of the present study was to examine the core areas of psychological interventions in adolescents with asthma. **Methods**: A scientific search of the literature was conducted, identifying 26 articles that described intervention programs targeting this population. **Results**: The findings show heterogeneity both in the characteristics of these interventions and in the variables assessed. Moreover, numerous studies do not clearly specify the theoretical framework employed, nor do they consider relevant factors such as comorbidity or the differing levels of disease severity. These limitations hinder the replicability and generalization of the results, as well as the understanding of psychological and behavioral impact. **Conclusions**: Therefore, the need to advance research that develops personalized programs integrating relevant aspects and improving quality of life and clinical outcomes is highlighted. Nevertheless, the interpretation of these findings is limited by the heterogeneity of the available evidence, including variability in intervention formats, theoretical frameworks, and outcome measures.

## 1. Introduction

Chronic diseases (CDs) represent one of the leading causes of mortality, reduced quality of life, and increased healthcare costs, with respiratory diseases being the third leading cause of death in 2021 [1]. Within the child and adolescent population, asthma represents the most prevalent chronic respiratory disease worldwide [2], affecting approximately 300 million people [3]. Its prevalence among adolescents aged 10–19 years is around 10.4% globally, which represents the developmental stage in which it occurs most frequently [4].

Asthma is characterized by inflammation and narrowing of the airways, resulting in breathing difficulties, wheezing, coughing, and chest tightness [5]. Although there is no cure, current treatments—mainly inhaled corticosteroids and bronchodilators—enable symptom control and improvement in quality of life. In addition, effective asthma management often includes trigger control and environmental remediation strategies aimed at reducing exposure to allergens and irritants [6].

Beyond its physical impact, asthma affects psychological well-being and overall quality of life [7]. A bidirectional relationship exists between physical and psychological well-being, whereby anxious–depressive symptomatology is associated with poorer functioning and lower treatment adherence [8]. During adolescence, asthma constitutes an additional vulnerability factor [9], as developmental changes may turn disease management into an additional stressor [10,11]. In this population, a higher risk of anxiety, depression, low self-esteem, and reduced quality of life has been identified, along with difficulties in disease control and therapeutic adherence [12,13,14]. Asthma may also generate insecurity in social and school settings, negatively affecting academic performance [9,10,15].

With regard to adherence, low rates and suboptimal disease control have been reported [11,16], influenced by factors such as low risk perception, limited knowledge, negative beliefs about medication, reduced self-efficacy, and emotional distress [17]. In turn, psychological distress may exacerbate asthma symptoms, with poorly controlled asthma being associated with school absenteeism, poorer academic performance, and social and emotional impairment [9,18].

Given the quality of life is closely linked to adherence and asthma control, promoting psycho-emotional health may contribute to improving clinical outcomes in adolescents with asthma [16]. In this regard, the utilization of psychological interventions has emerged as a potentially valuable component in the clinical management of asthma in children and adolescents [9]. These interventions address not only emotional distress but also key behavioral outcomes such as self-efficacy and medication adherence. Evidence suggests that interventions incorporating cognitive, behavioral, or psychosocial components may reduce symptoms of anxiety and depression, as well as improve asthma control, reduce the frequency of exacerbations, and increase treatment adherence, compared to usual care or treatments without a psychological component [19].

These interventions are commonly grounded in established models of health behavior change that have demonstrated efficacy, with social cognitive theory and the health belief model being frequently employed to develop intervention programs. These frameworks emphasize constructs such as self-efficacy, perceived benefits and barriers, and outcome expectations, which may influence adherence and self-regulation behaviors. The systematic application of theory has been demonstrated to clarify the mechanisms of change targeted by interventions and has been associated with enhanced effectiveness of digital asthma self-management programs. This highlights the importance of explicitly linking theoretical constructs to intervention components to optimize clinical outcomes and quality of life [20].

However, despite the extensive research on medical treatments, studies addressing the psychological impact of asthma during this developmental stage remain scarce [9]. Therefore, identifying key psychological indicators to inform the design of tailored interventions with solid theoretical foundations is considered essential. Given the multidimensional impact of asthma during adolescence, including physical as well as emotional, cognitive, and social challenges, there is a need for integrative constructs capable of capturing this complexity. In this relation, quality of life has been conceptualized as a dynamic, multidimensional construct that reflects individuals’ functioning across physical, emotional, cognitive, and social domains. More specifically, health-related quality of life (HRQoL) refers to the overall impact of the disease on these domains and daily functioning, with research showing significant impairments across these in adolescents with asthma compared with healthy peers [21]. Accordingly, integrative models of disease adjustment have emphasized the importance of jointly considering these areas to better understand adolescents’ adaptation to chronic illness and to guide the development of psychological interventions. Among these, the Disease Adjustment Model from an Integrative Perspective (DAMIP) provides a comprehensive framework for conceptualizing health-related quality of life during adolescence [22].

In this context, the aim of the present review is to review the scientific evidence on psychological intervention strategies targeting adolescents with asthma in relation to improving quality of life, disease coping strategies, and treatment adherence. Accordingly, this narrative review is guided by the following research question: “*Which psychological components and intervention strategies have been targeted in psychological interventions for adolescents with asthma over the last decade?*”.

## 2. Materials and Methods

A narrative (non-systematic) review of the scientific literature was conducted with the aim of analyzing psychological interventions in adolescents with asthma. This approach was selected to allow a broad and integrative synthesis of intervention strategies, study designs, and outcome domains, rather than to generate a comprehensive or exhaustive identification of all available studies.

The literature search was conducted using the Web of Science (WoS) (All Databases) and the Proquest Central databases at the University of Valencia. A structured search strategy was applied using the following terms: [psychol*] AND [“intervention” OR “treatment”] AND [adolesc*] AND [“asthma” OR “bronchial asthma” OR “allergic asthma”]. Broad search terms were intentionally applied to maintain the exploratory scope of the present review. Filters were applied to limit the results to peer-reviewed journal articles published in English or Spanish between 2015 and 2025. Only peer-reviewed journal articles were considered eligible for inclusion. Doctoral dissertations, conference proceedings, and other forms of gray literature were excluded.

The temporal restriction to studies published between 2015 and 2025 was implemented to capture recent developments in psychological interventions for adolescents with asthma, reflecting current clinical practices, theoretical perspectives, and the increasing emphasis on patient-centered approaches, quality-of-life outcomes, and digital or mobile health components. While technology-assisted interventions emerged prior to this period, earlier studies were often exploratory and less consistently integrated into routine clinical practice. Focusing on the past decade allowed the identification of interventions that are methodologically consolidated and clinically relevant to contemporary adolescent healthcare.

In addition, a supplementary manual search was conducted using Google Scholar to identify potentially relevant studies not retrieved through database searches. This complementary search followed an exploratory approach using the keyword combinations used in WoS and ProQuest related to psychological interventions, adolescence, and asthma, with titles, abstracts, and keywords screened for relevance according to the predefined inclusion criteria.

**Figure 1:**
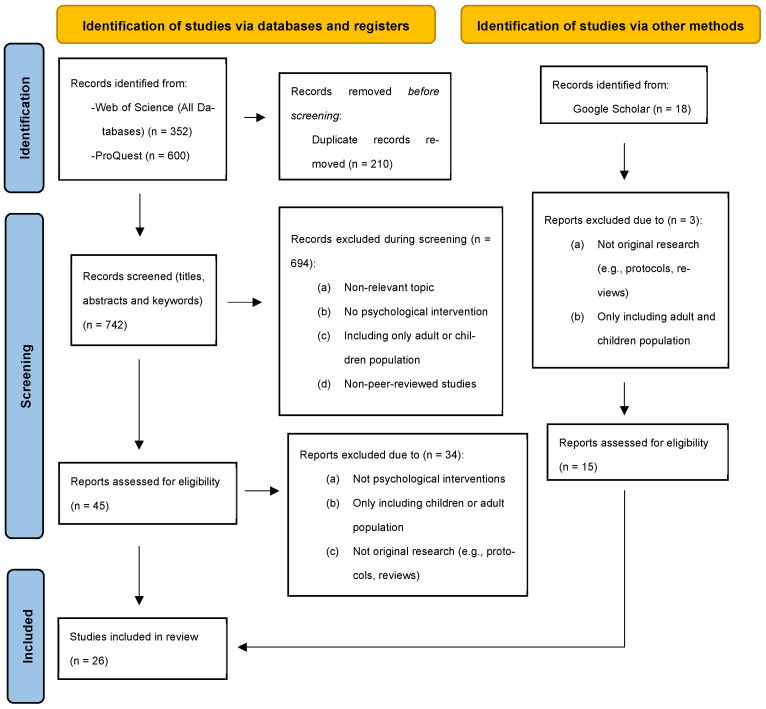
PRISMA flow diagram.

Following database searches, retrieved records were imported into a web-based screening platform (Rayyan QCRI) [23] to support reference management and duplicate identification. Therefore, duplicate records were identified and removed prior to screening. Subsequently, titles and abstracts were screened to assess relevance according to the predefined inclusion criteria. Figure 1 presents a PRISMA 2020 flow diagram [24], including a separate column for the manual search conducted via Google Scholar and detailed reasons for study exclusion to transparently describe the study identification and selection process.

Although a formal systematic review methodology was not adopted, several strategies were implemented to minimize selection bias. These included the use of predefined inclusion and exclusion criteria, the application of the same search strategy across all databases, independent screening of titles and abstracts by the authors, and consensus-based resolution of discrepancies during study selection.

### 2.1. Inclusion and Exclusion Criteria

The following criteria were applied in the selection of studies: (a) The interventions described in the articles included psychological components, defined as intervention elements targeting emotional processes, cognitive coping, self-efficacy, motivation, behavioral self-regulation, or psychosocial adjustment related to asthma management. (b) The articles included adolescents aged 12 to 16 years with asthma without other specified/related medical comorbidities (all studies included this age range, but not exclusively this age range). (c) The studies analyzed different psychological outcomes including self-regulation, self-management, self-efficacy, anxious–depressive symptomatology, health/illness-related quality of life, disease coping, and/or treatment/medication adherence. (d) The articles were published in a scientific journal. (e) The articles were published in English or Spanish. (f) The articles were published between 2015 and 2025. Mixed-methods studies were included only when they reported clearly defined quantitative outcomes directly aligned with the aims of the review, allowing for comparison with quantitative intervention studies.

Conversely, studies were excluded if they focused exclusively on (a) pharmacological or purely medical interventions, (b) children or adult populations, (c) observational or qualitative-only designs, (d) studies not reporting original empirical data (e.g., protocols), or (e) studies focusing only on non-asthma chronic conditions.

The age range of 12 to 16 years was selected to focus on early to mid-adolescence, a developmental period characterized by increasing autonomy in disease management while parental involvement remains relevant [25,26]. This range also reflects the most common age span reported across the included intervention studies, allowing for greater comparability of intervention targets, psychological processes, and outcome measures. Studies including broader age ranges were considered eligible when results for adolescents within this range were reported.

### 2.2. Study Selection and Synthesis Method

The initial search conducted through the Web of Science and ProQuest databases yielded a total of 952 articles. Titles and abstracts were screened for relevance, with keywords consulted only as a supplementary aid to support relevance assessment during abstract screening but were not used as a formal exclusion criterion. After excluding duplicates and studies that did not meet the inclusion criteria, a full-text review was carried out; in addition, a manual search was conducted using Google Scholar to identify potentially relevant studies not retrieved through the WoS and ProQuest database searches.

For all included studies, several aspects were examined, including sample size, intervention design, targeted psychological components, measurement instruments, theoretical justification, and quality-of-life indicators. To define and organize health-related quality-of-life (HRQoL) indicators, the Disease Adjustment Model from an Integrative Perspective (DAMIP) [22,27,28,29,30,31] was adopted as an analytical framework, given its integrative and theoretically grounded conceptualization of adjustment to chronic illness. This model synthesizes HRQoL indicators consistently identified across a wide range of theoretical approaches and empirical studies, offering a coherent structure applicable across heterogeneous intervention contexts.

In the present review, the DAMIP model was used to guide the interpretation of outcomes by mapping intervention targets and assessment measures onto a shared set of HRQoL domains, facilitating synthesis and comparison despite variability in study designs, intervention formats, and measurement instruments.

These health-related quality-of-life indicators are as follows:

- Physical well-being: physical health (including complaints or symptoms) and personal characteristics (e.g., age, height).
- Emotional well-being: emotional state and individual reactions (including stress, worries, etc.).
- Cognitive coping: capacity to cope with different adversities through the use of strategies.
- Social and support relationships: social interactions, including peer relationships and the family context.
- Identity: system of values and beliefs, as well as self-perception (both personal and physical) and aspects of the school environment (perceptions of academic abilities and feelings towards school).

Given the non-systematic and exploratory nature of the present review, no formal critical appraisal of study quality was conducted using standardized tools such as CASP, MMAT, or JBI. The primary objective of this review was not to compare effect sizes or determine the strength of evidence, but rather to map and synthesize the psychological components, theoretical frameworks, and quality-of-life indicators addressed in existing interventions for adolescents with asthma. Accordingly, the analysis focused on descriptive and conceptual aspects of the interventions rather than on methodological quality assessment. Nevertheless, relevant methodological characteristics of the included studies, such as study design, sample size, and intervention format, were systematically extracted and reported to provide contextual information for interpreting the findings.

## 3. Results

### 3.1. Basic Characteristics

As illustrated in Table S1 (included in the Supplementary Material), the paper presents a summary of the main characteristics of the 26 articles that were selected for the study. The earliest studies in this field were published in 2015 [32,33,34], while the most recent study was published in 2025 [35]. The year 2020 was characterized by the highest number of publications in the last decade (*n* = 6) (23.08%) [36,37,38,39,40,41], with 2017 [42] and 2025 [35] being the years with the lowest scientific output (*n* = 1) (3.85%). Within the selected databases and according to the applied inclusion criteria, a conspicuous absence of records from 2016 and 2023 was observed.

With regard to the inclusion criteria employed in the various studies, the majority of the articles specified the type of asthma from which the participants suffered. The prevalence of persistent asthma was found to be the most common form, with 11 out of the total number of cases (42.31%) falling into this category. In addition, four studies (15.38%) permitted adolescents with persistent and/or uncontrolled or poorly controlled asthma to participate. In a similar vein, 11.54% of the included studies (*n* = 3) reported samples composed exclusively of adolescents with uncontrolled asthma, with no additional clinical characterization reported. In contrast, nine articles (34.61%) limited themselves to noting the presence of a general diagnosis of asthma, without specifying any clinical subtype; one of them (3.85%) specixfied, however, that the diagnosis should be recent. A mere 3.85% (*n* = 1) of the studies included participants with acute asthma, and another 3.85% (*n* = 1) specifically focused on allergic asthma.

From a geographical perspective, the majority of the research was conducted in the United States (*n* = 17) (65.38%), although studies were also identified from Iran (*n* = 2) (7.14%), New Delhi (*n* = 1) (3.57%), the Netherlands (*n* = 1) (3.85%), Turkey (*n* = 1) (3.57%), Greece (*n* = 1) (3.57%), Puerto Rico (*n* = 1) (3.57%), Taiwan (*n* = 1) (3.57%), and China (*n* = 1) (3.57%).

Regarding sample size, there was substantial variability across the included studies, ranging from 13 to 430 participants [43,44]. Most studies included relatively small to moderate samples.

### 3.2. Methodological Characteristics

In terms of intervention format, fifteen studies (57.69%) implemented in-person interventions, nine studies (34.61%) delivered interventions online, one study (3.85%) adopted a hybrid format—combining both types of delivery—and another one (3.85%) delivered in-person intervention through home visits, combining it with support phone calls.

The duration of the interventions demonstrated considerable heterogeneity, ranging from short-term programs—with an approximate duration of three weeks—to prolonged interventions lasting six months. The mean duration of the programs analyzed was approximately ten weeks.

In relation to study design, the majority of studies employed randomized designs, primarily randomized controlled trial or randomized pilot study formats (*n* = 11) (42.31%). In addition, several specific randomized designs were identified, including randomized controlled trials with repeated measures (*n* = 1) (3.85%), cluster randomized controlled trials (*n* = 1) (3.85%), and parallel randomized controlled trials (*n* = 2) (7.69%). Other methodological approaches included a multicenter study (*n* = 1) (3.85%), a mixed-methods multi-method trial (*n* = 1) (3.85%), a randomized comparative study (*n* = 1) (3.85%), a single-group pretest–posttest pilot study (*n* = 1) (3.85%), and two pretest–posttest feasibility studies (*n* = 2) (7.69%).

### 3.3. Study Variables

The analysis revealed substantial heterogeneity among the variables examined. The most frequently examined were asthma control (*n* = 16) (61.54%), disease management abilities (*n* = 9) (34.61%), asthma-related self-efficacy (*n* = 8) (30.77%), and illness-related quality of life (*n* = 8) (30.77%). In addition, treatment adherence (*n* = 4) (15.38%), medication adherence (*n* = 6) (23.08%), and health-related quality of life (*n* = 1) (3.85%) were also identified as areas of focus. At a familiar level, disease management (*n* = 2) (7.69%), family communication (*n* = 1) (3.85%), family functioning (*n* = 1) (3.85%), and family empowerment (*n* = 1) (3.85%) were also analyzed, although less frequently. While these latter variables are studied within the family unit, the adolescent is also included in this context, with family functioning being also able to exert a significant influence on the adolescent’s quality of life and their ability to adapt to disease challenges.

In the field of educational components—which primarily addressed asthma knowledge, medication purpose and use (e.g., inhaler technique), trigger identification, and beliefs about treatment—the following variables were included: beliefs about medication and adherence (*n* = 3) (11.54%), beliefs about the disease (*n* = 1) (3.85%), and asthma knowledge (*n* = 5) (19.23%).

Furthermore, certain studies focused on variables associated with the direct manifestations of the disease, including the perception of threat (*n* = 2) (7.69%), perceived severity (*n* = 3) (11.54%), interference with daily life (*n* = 1) (3.85%), problems with medications (*n* = 1) (3.85%), asthma triggers in the home environment (*n* = 1) (3.85%), the presence of asthma and allergic rhinitis symptoms (*n* = 1) (3.85%), school absenteeism (*n* = 2) (7.69%) and school attendance (*n* = 1) (3.85%), and sense of importance and motivation to take medication (*n* = 1) (3.85%).

In relation to psychological variables, the included studies evaluated anxiety symptoms (*n* = 5) (19.23%), depressive symptomatology (*n* = 6) (23.08%), psychological flexibility (*n* = 1) (3.85%), general mood and behavioral symptoms (*n* = 1) (3.85%), post-traumatic symptoms (*n* = 1) (3.85%), self-regulation (*n* = 2) (7.69%), perceived stress (*n* = 3) (11.54%), coping skills in stressful situations (*n* = 1) (3.85%), social support (*n* = 1) (3.85%), problem-solving skills (*n* = 1) (3.85%), and personal beliefs (*n* = 1) (3.85%).

Finally, the following additional variables were considered: expectations about treatment outcomes (*n* = 2) (7.69%); the existence of a children’s asthma action plan (*n* = 1) (3.85%), reflecting ownership of a self-management tool following educational intervention; reasoning in different asthma-related scenarios (*n* = 1) (3.85%); physical activity (*n* = 1) (3.85%); self-efficacy for exercise (*n* = 1) (3.85%); diet quality (*n* = 1) (3.85%); sleep quality (*n* = 1) (3.85%); fatigue (*n* = 1) (3.85%); and pain (*n* = 1) (3.85%).

### 3.4. Theoretical Basis

The reporting of theoretical frameworks was examined as a descriptive characteristic of the interventions to assess the extent to which existing programs were theoretically grounded, rather than as an inclusion criterion.

Of the articles analyzed in this study, a considerable proportion did not specify the existence of a theoretical framework to support the design or justification of the intervention implemented (*n* = 11) (42.31%). Of these, only one study (3.85%) mentioned having used a theoretical model as a basis, although without specifying which one.

Among the studies that did explicitly describe a theoretical basis, three (11.54%) used social cognitive theory as their main reference, two (7.69%) relied on Leventhal’s Common Sense Model of Self-Regulation, and two additional studies (7.69%) used the theoretical framework of pediatric self-management proposed by Modi et al. (2012) [45]. Similarly, one study (3.85%) was grounded in the Socioecological Model, another (3.85%) in the Transtheoretical Model, one (3.85%) in the Asthma Acceptance Model, and a final one (3.85%) in Bandura’s Self-Efficacy Model.

Finally, several studies incorporated intervention principles based on Motivational Interviewing (*n* = 4) (15.38%) or Cognitive Therapy (*n* = 1) (3.85%). The remaining interventions that reported some type of theoretical basis were supported by general guidelines issued by international organizations or specialized health institutions (*n* = 5) (10.23%).

### 3.5. Quality-of-Life Indicators

By taking the quality-of-life indicators proposed by the DAMIP model [22] as a point of reference, it is observed that the majority of the studies reviewed concentrate primarily on dimensions of physical well-being as the basis for developing interventions. These components were addressed primarily through educational programs aimed at providing information about asthma and its clinical management, with the goal of improving understanding of the disease and treatment adherence. While some studies explicitly evaluated health- or disease-related quality of life, most did not measure these constructs directly, instead addressing related outcomes such as asthma control, medication adherence, or disease management abilities.

In addition, 53.85% of the studies incorporated elements related to cognitive coping (*n* = 14), emphasizing goal setting, problem solving, and the implementation of self-regulation strategies aimed at optimizing disease management and the perception of self-control.

With regard to the emotional dimensions of well-being, only five studies (19.23%) included content aimed at exploring and addressing the concerns of both adolescents and their families, mainly addressing anxiety–depressive symptomatology and emotional management associated with asthma.

In terms of personal identity, a mere two programs (7.69%) addressed this component, incorporating reflections on individual values and beliefs and their influence on the experience of illness.

Finally, five interventions (19.23%) included aspects related to social and support relationships, with the aim of strengthening family communication and, in some cases, promoting better coordination with the school and social environment.

## 4. Discussion

The aim of the present review was to increase awareness about psychological interventions for adolescents with asthma in order to identify the key variables that have been studied and targeted. The scientific evidence reveals a scarcity of specific psychological interventions, despite the substantial emotional and social impact associated with the condition. Most of the reviewed interventions primarily focused on aspects such as treatment adherence or health education, while essential psychological components—including emotional regulation, self-efficacy, and coping—were largely overlooked, despite their fundamental role in clinical outcomes and quality of life in this population [40,46,47,48,49]. These findings highlight the need to consider psychological well-being as a central therapeutic objective rather than merely a secondary outcome.

In addition, a lack of robust theoretical frameworks guiding the design and evaluation of interventions was identified. An explicit theoretical foundation would facilitate the selection of intervention goals and strategies, the identification of mechanisms of change, and the replicability and generalizability of findings [50,51,52]. This need becomes particularly salient in emerging fields such as mHealth interventions, where a sound theoretical basis is critical to link digital components to specific psychological processes and to determine whether observed changes are genuinely attributable to the proposed mechanisms or instead to factors related to the application’s design, accessibility, or usability [53].

Another relevant issue is the limited consideration of asthma-related comorbidities, despite the fact that asthma commonly coexists with other chronic conditions such as allergic rhinitis, atopic dermatitis, or food allergies, among others [54,55,56]. Ignoring this complexity constrains the understanding of psychological and behavioral impact in more complex clinical scenarios, where the presence of multiple symptoms or treatments may influence perceived control, treatment adherence, and emotional well-being. Similarly, study outcomes rarely differentiate between levels of disease severity [57], paying little attention to intermittent or acute asthma, which may be associated with heightened psychological distress and greater limitations in daily functioning.

As highlighted throughout this review, the included interventions showed substantial heterogeneity in format, duration, methodological design, psychological components, outcome measures, and theoretical basis. While this diversity reflects the complexity of asthma management during adolescence and the evolving nature of intervention approaches, it also limits the interpretability and generalizability of findings. The use of diverse assessment tools and heterogeneous samples, including variability in asthma severity, comorbidities, and contextual factors, complicates comparison across studies and restricts conclusions regarding the relative effectiveness of specific intervention components.

In consideration of the aforementioned points, the present review contributes to updating the available scientific knowledge on psychological interventions targeting adolescents with asthma over the past decade, offering a structured synthesis that may serve as a foundation for improving future therapeutic approaches. Nevertheless, certain methodological limitations related to the scope of the review should be acknowledged, including the limited number of studies analyzed, the temporal delimitation applied, and the use of only three databases, excluding other relevant databases such as PubMed and CINAHL. These factors may have affected the comprehensiveness of the findings. However, Web of Science and ProQuest index a substantial proportion of journals also covered by these databases, particularly in the fields of psychology, behavioral health, and interdisciplinary health research. Therefore, although some studies may have been missed, the selected databases are expected to have captured a significant share of the relevant peer-reviewed literature. Accordingly, given its narrative nature and the limited number of databases consulted, the present review should not be interpreted as a comprehensive synthesis of the literature.

Accordingly, the need for greater conceptual and methodological consistency in future research, including clearer reporting of intervention components, the use of standardized outcome measures, and the adoption of explicit theoretical frameworks, is underscored throughout the aforementioned heterogeneity. In this regard, theory-based psychological interventions may facilitate the identification of target variables and mechanisms of change, enhance replicability, and support the development of more effective digital and mHealth interventions. Moreover, future studies should prioritize the systematic examination of mechanisms of change, using mediation analysis or longitudinal designs, to better understand how psychological processes influence adherence, disease control, and quality of life and optimize the effectiveness of interventions and more personalized approaches in adolescents with asthma.

## 5. Conclusions

While the present narrative review does not provide a comprehensive synthesis of all available evidence, it offers an integrative overview of recent psychological interventions for adolescents with asthma and highlights consistent gaps in their psychological scope and theoretical grounding. The findings identify recurring patterns and unmet needs that may inform future research and intervention development. Accordingly, these results should be interpreted as indicative rather than definitive, underscoring the need for more systematic and theoretically grounded studies in this area.

## Data Availability

No new data were created or analyzed in this study.

## Supplementary Materials

The following supporting information can be downloaded at https://www.mdpi.com/article/10.3390/children13020181/s1, Table S1: Psychological interventions for asthma.
