# Supplementary material for: Psychological Intervention Strategies in Adolescents with Asthma: A Review of the Literature
Source: Children (Basel). 2026 Jan 28;13(2):181. doi: 10.3390/children13020181 (PMC12939749; doi:10.3390/children13020181)
Supplement: Supplementary file 1 [file children-13-00181-s001.zip › children-4045046-supplementary.pdf]

**Table S1. Psychological interventions for asthma.**

| Author(s) | Year and Country | Asthma variant | Sample | Age | Study design | Intervention | Variables and assessment tools | Theoretical basis | Format | Duration | Quality-of-life indicators |
|---|---|---|---|---|---|---|---|---|---|---|---|
| Barikani et al. | 2021 (Iran) | Moderate–severe persistent asthma | 52 adolescents | 10 – 18 years | Randomized controlled trial | Participants in both groups received standard medical treatment for asthma, comprising regular medical visits and prescriptions. Patients in the experimental group also received three additional one-hour Motivational Interviewing (MI) sessions each week.<br>The intervention was grounded in the principles of MI, and it comprised three weekly one hour-educational sessions, which were conducted individually and focused on treatment adherence, beliefs about medications, and self-efficacy. | **Medication adherence** → *Ten-Item Medication Adherence Scale (MARS)*<br><br>**Self-efficacy** → *CASES Questionnaire*<br><br>**Beliefs about medication** → *BMQ Questionnaire* | Social Cognitive Theory<br><br>Motivational Interviewing | In-person | Three sessions of 1 hour per week (total duration of 3 weeks) | Physical wellbeing<br><br>Cognitive coping<br><br>Identity |
| Bruzzese et al. | 2021 (USA) | Uncontrolled asthma | 61 adolescents | Mean age 15,2 years | Randomized pilot study | The intervention group received the CAMP Air Intervention (Controlling Asthma Program for Adolescents), which is an e-health program developed with the objective of enhancing the outcomes of asthma in adolescents. It consists of seven modules, each addressing fundamental educational subjects and incorporating personalized sessions. It is complemented by integrated interactive practical activities, personalized feedback, and dynamically generated supplementary resources, all of which are designed to cater to adolescents experiencing uncontrolled asthma.<br><br>The control group was provided with educational material that had been utilized in their prior studies on asthma and stress. | **Asthma knowledge** → 40-item questionnaire of true/false questions<br><br>**Asthma control** → *Asthma Control Test (ACT)*<br><br>**Symptoms severity within the last two weeks** → *Whalgren's Asthma Symptom Scale*<br><br>**Quality of life related to illness** → *Pediatric Asthma Quality of Life Questionnaire (PAQLQ)* | Social Cognitive Theory and Motivational Interviewing | Online | CAMP Air group → seven modules with one-week gap between each module<br><br>Control group → one session of orientation of 15 minutes | Physical wellbeing<br><br>Emotional wellbeing |
| Cushing et al. | 2019 (USA) | Persistent asthma | 20 adolescent–caregiver dyads | Adolescents between 13 – 17 years and their caregivers | Multicenter study | They received the Responsive Asthma Care for Teens (ReACT) intervention. It is an adaptative mHealth intervention whose purpose is to facilitate self-regulation and self-control, goal setting, and problem solving in adolescents. | **Asthma knowledge and abilities** → *Asthma Child Knowledge and Skills Questionnaire*<br><br>**Asthma control** → *Asthma Control Test*<br><br>**Asthma management** → *Asthma Management Efficacy Questionnaire*<br><br>**Adherence to treatment** → *Medication Adherence Report Scale for Asthma*<br><br>**Self-regulation** → *Treatment Self-Regulation Questionnaire—Asthma*<br><br>**Stress** → *Adolescent Stress Questionnaire*<br><br>**Social support** → *Social Support Questionnaire*<br><br>**Social problem solving** → *Social Problem Solving Inventory-Revised: Short Form*<br><br>**Quality of life related to asthma** → *PAQLQ* | It is asserted that a theoretical foundation underpins this approach; however, this basis remains unidentified and unexpounded. | Online | | Physical wellbeing<br><br>Cognitive coping |
| Davis et al. | 2019 (USA) | Persistent asthma | 319 adolescents | 11 – 17 years | Randomized controlled trial | The experimental group was presented with a brief video on the management of asthma and was subsequently administered a one-page questionnaire.<br><br>The control group received only the standard care that is typically provided for this condition. | **Asthma management from adolescents and caregivers**<br><br>**Caregivers and adolescents' self-efficacy**<br><br>**Caregivers and adolescents' expectations**<br><br>**Medication adherence** → Visual Analog Scale (VAS) measure of adherence<br><br>**Problems related to medication** → questions adapted from the Brief Medication Questionnaire to be specific to asthma | Social Cognitive Theory | In-person | | Physical wellbeing |
| Fedele et al. | 2021 (USA) | Persistent and poorly controlled asthma | 33 adolescent–caregiver dyads | 12 – 15 years | Randomized controlled trial | The experimental group received the AIM2ACT intervention, which is a mobile app focused on asthma control. It has been developed to assist in the identification of a goal related to disease control, and to establish a behavioral contract between the dyads. The contract sets out specific steps to achieve the aforementioned goal, as well as the timeframe for completion and the reward to be received upon completion. At the conclusion of the designated period for achieving the aforementioned objective, the app prompts the user to assess their progress and engage in problem-solving communication.<br><br>The dyads in the control group received usual care and performed analogous exercises, although in paper format. | **Family illness management** → *Family Asthma Management System Scale (FAMSS)*<br><br>**Asthma control** → *ACT*<br><br>**Asthma-related quality of life** → *Pediatric Asthma Quality of Life Questionnaire*<br><br>**Pulmonary function** (FEV1)<br><br>**Asthma management self-efficacy** → *Asthma Management Efficacy Questionnaire*<br><br>**Family communication** → *Decision Making Involvement Scale (DMIS (decision-making subscale)* | Theoretical framework for pediatric self-management by Modi et al., 2012 | Online | 20 weeks of duration (4 months of follow up) | Physical wellbeing |

| | | | | | | | | | | | |
|---|---|---|---|---|---|---|---|---|---|---|---|
| Goel et al. | 2015<br>(New Delhi) | Persistent asthma | 40 dyads | 7 – 12 years | Randomized controlled trial with repeated measures | The experimental group received a theoretical and practical training intervention delivered by an asthma educator. At the end, the child was invited to formulate two to three goals that they would like to achieve prior to the next visit.<br><br>In the "usual care" group, which functioned as the control group, parents and children received a standard information pack when they attended the doctor for a routine visit. | **Caregiver quality of life** → Pediatric asthma caregiver quality of life Questionnaire (*PACQLQ)*<br><br>**Caregiver asthma knowledge** → *customized Asthma Knowledge Questionnaire (AKQ)*<br><br>**Children asthma control** → *Asthma Control Questionnaire (ACQ)*<br><br>**Beliefs about medication and adherence** → *Brief Medication Questionnaire (BMQ)*<br><br>**Children action plan about asthma** → *Written asthma action plan (WAAP)* | This intervention was designed based on key principles of pedagogy and health education and was named the "Sehatmand Saanse Programme (Healthy Breathing Programme)".<br><br>The Global Initiative for Asthma (GINA) guidelines served as the basis for the asthma education content. | In-person | 6 months | Physical wellbeing |
| Halterman et al. | 2022<br>(USA) | Persistent or poorly controlled asthma | 430 adolescents | 12 – 16 years | Randomized clinical trial (three arms, parallel) | Two intervention groups:<br><br>- SB-ACT group (School-Based Asthma Care for Teens) (DOT + MI assessment) → SB-ACT includes the following components: (1) directly observed therapy (DOT) of preventive medications at school to allow adolescents to experience the potential benefits of following guideline-based asthma treatment and (2) a counseling intervention with Motivational Interviewing that included asthma education and support for adolescents to transition to independent long-term treatment adherence. The ultimate goal of SB-ACT was to support adolescents' transition to independent and sustained use of preventive medication. The MI component consisted of an evidence-based self-management program to support the adolescent in their transition to independence with preventive medication use. The goal of the counseling sessions was to help adolescents move toward autonomy with medication and decision-making.<br><br>- DOT-only group → these adolescents received 6-8 weeks of directly observed administration of asthma medication by the school nurse but did not receive MI assessment.<br><br>The control group (asthma education (AE)) received usual medication care and an asthma education program at school. This program included similar content to the MI assessment part of SB-ACT. | **Medication adherence** → answering questions like "During the last two weeks, I took my control medication…" with answer options from "nothing" to "everyday".<br><br>**Sense of confidence, importance and motivation to take medication** → 10-point rating scale<br><br>**Scholar absenteeism**<br><br>**Quality of life** → *Pediatric Asthma Quality of Life Questionnaire & Pediatric Asthma Caregiver's Quality of Life Questionnaire* | Motivational Interviewing (MI) | In-person | Group SB-ACT → MI assessment started 4-6 weeks after the DOT. The three MI sessions consisted of an initial session of assessment of 30-40 minutes (4-6 weeks after DOT start) and 2 sessions of follow-up of 20-30 minutes 2 and 6 weeks after.<br><br>DOT group → between 6 and 8 weeks<br><br>Control group → three educative sessions | Physical wellbeing<br><br>Cognitive coping |
| Horner et al. | 2018<br>(USA) | Asthma (and weight problems) | 13 children and adolescents and their parents | 9 – 14 years | Pretest–posttest feasibility study (single group) | The experimental group that participated in the *"Living Healthy with Asthma"* intervention was family-based and combined daily health promotion with a focus on weight management and asthma self-management. This intervention addressed both asthma self-management and healthy lifestyle habits for weight control at each visit.<br><br>The control group received the same education and materials for asthma self-management as the experimental group. The content in this case was educational. | **Asthma severity** → *Three-item Severity of Chronic Asthma Scale*<br><br>**Asthma self-management** → *Asthma Inventory for Children*<br><br>**Physical activity self-efficacy** → *Child Physical Activity Self-Efficacy Scale*<br><br>**Children quality of life** → *Pediatric Asthma Quality of Life Scale*<br><br>**Parents quality of life** → *Pediatric Asthma Caregiver's Quality of Life Scale*<br><br>**Diet quality** | Motivational Interviewing (MI) | In-person and by telephone (home visits and with support calls) | 12 weeks in six home visits and three support telephone calls | Physical wellbeing<br><br>Cognitive coping |
| Joseph et al. | 2018 (USA) | Acute asthma | 121 adolescents | 13 – 19 years | Randomized controlled pilot study | *"Puff City Asthma Management Program"* is an online program consisting of an initial survey and four educational sessions using adapted computers. The sessions addressed asthma control as well as psychosocial issues such as smoking, depression, perceived emotional support, and lack of health insurance or a regular family doctor.<br>The program focused on three main aspects: medication adherence, having a rescue inhaler on hand, and reducing or quitting smoking. | **Asthma control** → *Asthma Control Test* (ACT)<br>**Asthma-related self-regulation** → *Asthma Self-Regulation Development Interview* (ASRDI). | One of the main theories used in the program is the Transtheoretical Model (TTM) | Online | Each interactive session lasted between 15 and 30 minutes. A booster session is also included after 6 months. | Physical wellbeing<br><br>Cognitive coping<br><br>Social and support relationships |
| Karatas & Calisir | 2024<br>(Turkey) | Allergic asthma | 41 adolescents | 10 – 14 years | Randomized controlled trial (non-blind) | The two experimental groups received standard medical care and education from other adolescents (peer group) or adults.<br><br>The control group received usual hospital care but did not receive training. | **Anxiety symptoms** → *State Anxiety Inventory for Children* (SAIC)<br><br>**Asthma-related self-efficacy** → *Self-Efficacy Scale for Children and Adolescents with Asthma* (SES)<br><br>**Asthma-related quality of life** → *Pediatric Asthma Quality of Life Questionnaire* (PAQLQ)<br><br>**Knowledge and management of disease** → *Disease Knowledge and Management Questionnaire* (DKMQ) | Not specified | Online | The two experimental groups received six weekly sessions of education on allergic asthma on different days of the week.<br>The sessions lasted between 20 and 30 minutes. | Physical wellbeing |

| | | | | | | | | | | | |
|---|---|---|---|---|---|---|---|---|---|---|---|
| Kintner et al. | 2015 (USA) | Asthma | SHARP intervention → 94 dyads<br><br>OAS intervention → 74 dyads | 9 – 14 years | Randomized controlled cluster trial (single-blind, prospective, two cohorts) | One group received the SHARP intervention (*Staying Healthy – Asthma Responsible & Prepared*) which is a program that has two components: one for students at school and the other for members of the students' social network in the community (the school component is the focus of this study).<br><br>The other group participated in the OAS intervention (*Open Airways for Schools*), which is a well-established non-academic program on asthma education in schools. Students in this group received six 50-minute classes and took home informational brochures. | **Medical history** → *General Health History Survey (GHHS)*<br><br>**Asthma knowledge** → *Knowledge of Asthma Survey (KAS)*<br><br>**Reasoning about different scenarios related to asthma** → *Reasoning about asthma scenarios (RAA)* | The SHARP intervention is based on the Asthma Acceptance Model (AAM), developed from an ecological approach within a lifelong development perspective. | In-person | SHARP intervention → 10 sessions of 50 minutes each.<br><br>OAS group → six classes of 50 minutes each. | Physical wellbeing<br><br>Social and support relationships |
| Kosse et al. | 2017 (The Netherlands) | Persistent asthma | 234 adolescents | 12 – 18 years | Randomized controlled cluster trial | The experimental group received the ADAPT intervention (*Adolescent Adherence Patient Tool*), which consisted of an interactive mobile app designed to improve adherence to asthma medication through questionnaires to monitor symptoms and adherence, medication reminders, short videos on different asthma-related topics, and chat with the pharmacist and peers.<br><br>The control group only received usual care. | **Health-related quality of life** → *RAND-36*<br><br>**Asthma and allergic rhinitis symptoms** → *Control of Allergic Rhinitis and Asthma Test (CARAT)*<br><br>**Asthma-related quality of life** → *Pediatric Asthma Quality of Life Questionnaire*<br><br>**Asthma control** → Asthma control questionnaire (ACQ)<br><br>**Illness perception** → *Brief Illness Perception Questionnaire*<br><br>**Medication beliefs** → *Beliefs about Medicines Questionnaire-Specific*<br><br>**Medication adherence** → *MARS* | The intervention is based on the Leventhal's *Common Sense Model of Self-Regulation* (CSM). | Online | Adolescents were given access to the intervention for 6 months. | Physical wellbeing |
| Koumpagioti et al. | 2020 (Greece) | Newly diagnosed asthma (all of them experienced at least two moderate/severe exacerbations requiring corticosteroids during the 12 months prior to referral) | 78 children and adolescents | 4 – 16 years | Randomized clinical trial (two groups, parallel) | The experimental group received an intervention in which children/adolescents and their carers had to attend an educational program on asthma care focused on developing self-management skills and promoting personal responsibility and self-efficacy when dealing with asthma-related problems. | **Treatment adherence** → Smart inhalers<br><br>**Pulmonary function**<br><br>**Asthma control** → Asthma Control Test (ACT) | Not specified | In-person | The monitoring lasted 6 weeks and the program lasted one interactive session of 45-60 minutes. | Physical wellbeing |
| Lin et al. | 2020 (USA) | Uncontrolled asthma | 21 participants | 10 – 17 years | Pretest–posttest feasibility study (single group) | During the study period, participants attended seven monthly medical appointments with an asthma specialist (pediatric pulmonologist or allergist) and five self-management school visits with a psychologist specializing in adherence.<br><br>Based on medical assessment (after four weeks of initial evaluation), a total of five self-management visits were scheduled every two weeks. During the self-management visits, the psychologist reviewed adherence data with the adolescent, identified and discussed barriers to adherence, discussed the relationship between medication adherence and asthma control, engaged in problem solving to select strategies to improve adherence, and provided personalized supportive text messages and reminders. | **Asthma severity** → *Composite Asthma Severity Index (CASI)*<br><br>**School absenteeism rate**<br><br>**Use of asthma-related health services**<br><br>**Asthma control** → *Asthma Control Test (ACT)*<br><br>**Medication adherence** → real-time measurement with Propeller Health | Not specified | Online | The study lasted 6 months. | Physical wellbeing<br><br>Cognitive coping |
| Marsland et al. | 2018 (USA) | Persistent asthma | 104 children and adolescents | 8 – 14 years | Randomized parallel controlled trial | The first group received the "*I Can Cope* (ICC)" intervention. This is a manualized intervention consisting of six individual sessions and a workbook for children focused on training them to manage asthma-related stress. Children received audio relaxation exercises on CD or MP3 files and a portable CD player. The also received points ("coping coins") for paying attention, participating in the session, and completing assigned tasks. These coins could be exchanged for small prizes (e.g., pencils, notebooks, etc.) at each session or saved for larger prizes (e.g., games, small toys, etc.).<br>Parents received information about the content of the sessions and, in addition, a phone call or text message after each session to review the material and their child's progress, reinforce their participation, and encourage them to ask questions.<br><br>The second group received the Open Airways for Schools (OAS) program (a modified version of the American Lung Association (ALA) Open Airways for Schools program). This program included six 40-minute sessions covering the NHLBI recommendations for asthma education. It focused on asthma education but also included information on stress management.<br><br>Finally, the NT group did not receive any treatment until after the trial, when they were offered the OAS program. | **Children's psychological state** → *Perceived Stress Scale (PSS-10); Child Depression Index (CDI); State-Trait Anxiety Inventory for Children (STAI-C); Children's Health Survey for Asthma (CHSA (parent version) and CHSDA-C (child version))*<br><br>**Children's sleep quality** → a diary-style record with two questions to be scored daily on a 10-point Likert scale ("Last night, the quality of my sleep was…" and "This morning, my awakening was…")<br><br>**Asthma-related medical measures** (e.g., lung function) | Not specified | In-person | ICC → 6 individual sessions of 50 minutes each<br><br>OAS → 6 sessions of 50 minutes each | Physical wellbeing<br><br>Emotional wellbeing<br><br>Cognitive coping |

| | | | | | | | | | | | |
|---|---|---|---|---|---|---|---|---|---|---|---|
| Martin et al. | 2015 (USA) | Persistent or uncontrolled asthma | 93 participants completed the 12 months follow-up | 5 – 18 years | Randomized controlled trial | The CURA Project was a trial to evaluate the effectiveness of an intervention with community health workers (CHWs) to improve asthma outcomes in children and adolescents. Based on that study, the present study hypothesized that the CHW group would improve medication adherence and reduce asthma triggers in the home compared to a control group receiving standard care.<br><br>The intervention was carried out by a community health worker and consisted of receiving general information about asthma, control and quick-relief medications, inhalers and spacers, symptom recognition, asthma triggers, and access to medical care. Families were provided with written information about where they could receive medical care, insurance, housing assistance, and home rehabilitation. Self-management skills were also addressed, including reorganizing the environment, problem solving, seeking social support, and self-monitoring.<br>At the end of each home visit, families completed a behavioral change plan in which they wrote down one small change they wanted to make in the following month.<br><br>The control group received postcards with a written version of the asthma content (following the same schedule as the experimental group). | **Asthma triggers at home** → self-reports, visual assessment, and objective measurements<br><br>**Asthma control** → based on the guidelines in the Expert Panel 3 Report, questions about symptoms, medication use, and missed activities were used. The Asthma Functional Severity Scale was also used to assess asthma control during the previous 12 months.<br><br>**Depressive symptoms** → PHQ-9<br><br>**Perceived stress** → Perceived Stress Scale (for caregivers) and Life Events Checklist (for children) | Not specified | In-person | CHW group participants were offered four home visits over 4 months | Physical wellbeing<br><br>Emotional wellbeing<br><br>Cognitive coping |
| McGovern et al. | 2019<br>(USA) | Persistent asthma | 33 children and adolescents, and their caregivers | 8 – 12 years | Pretest–posttest pilot study, single group | Children and adolescents participated in the COPE Intervention (*Creando oportunidades para el empoderamiento personal para el asma*). This was a seven-session CBSB intervention for children and adolescents with symptoms of anxiety and depression (in this case adapted for asthma: "COPE for Asthma"). Its aim was to improve self-efficacy and the ability to cope with stress and challenges associated with asthma, thereby reducing symptoms of anxiety and depression. It also helped children develop and use cognitive–behavioral skills (e.g. changing negative thoughts, using positive self-talk, activities for living in the moment, goal setting and problem solving, etc.). | **Anxiety symptoms** → *Screen for Child Anxiety-Related Emotional Disorders (SCARED)*<br><br>**Depressive symptoms** → *Patient-Reported Outcome Measurement Information System (PROMIS) Short Form for Depressive Symptoms*<br><br>**Asthma-related management and self-efficacy** → *Child Asthma Management Self-Efficacy (CASE)*<br><br>**Personal beliefs** → *Personal Beliefs Scale – Child Version (PBS-C)*<br><br>**Asthma symptoms** → *Childhood Asthma Symptom Checklist (CASCL)*<br><br>**Illness perception** → *Asthma Illness Representation Scale – Child Version (AIRS-C)*<br><br>**Illness-related quality of life** → *Pediatric Asthma Quality of Life Questionnaire (PAQLQ)*<br><br>**Disease interference** → *Childhood Asthma Control Test (C-ACT)* | The conceptual model of this study integrates a cognitive–behavioral skill building (CBSB) intervention into Leventhal's Common Sense Model (CSM) of illness representation and cognitive therapy (CT). | In-person | The study lasted two months.<br>The intervention sessions were held once a week for seven weeks.<br>These were group sessions (small groups of five students or fewer).<br>Each session lasted 30 minutes. | Physical wellbeing<br><br>Emotional wellbeing<br><br>Cognitive coping |
| Naar et al. | 2018<br>(Puerto Rico) | Persistent asthma | 167 families | 12 – 16 years | Randomized trial | Families in the experimental group participated in an intervention which consisted of MST-HC adapted for the treatment of poor self-management in adolescents with asthma. MST therapists began with an initial motivational session to promote commitment, followed by a functional assessment of asthma self-management based on the socioecological model (this assessment included interviews and in vivo observations in the home and community to identify triggers, antecedents, and consequences of inadequate asthma self-management in the child, family, school, community, and medical health systems). Based on this assessment, the MST therapist chooses from several evidence-based interventions that can be used to best address the identified behavioral problems.<br><br>Families assigned to the control group (FS intervention) received weekly, home-based, client-centered, non-directive family counseling. | **Pulmonary function**<br><br>**Medication adherence** → *Medication Adherence subscale of the Family Asthma Management System Scale (FAMSS); Daily Phone Diary (DPD)*<br><br>**Number of hospitalizations and medical visits related to asthma** | Socioecological Model | In-person | MST-HC group → treatment was planned to last up to 6 months after initiation. Sessions could take place several times a week at the beginning and then only once a week (once the adolescent's asthma control was improved)<br><br>FS group → 6-month duration. One weekly session of approximately 45 minutes was chosen (consistent with traditional outpatient therapy approaches) | Physical wellbeing<br><br>Social and support relationships |

| | | | | | | | | | | | |
|---|---|---|---|---|---|---|---|---|---|---|---|
| Nichols et al. | 2020 (USA) | Asthma (and obesity) | 30 adolescents with asthma and obesity | Adolescents between 10-17 years | Multimethod study, mixed approach (qualitative and quantitative) | The participants received a nurse-led intervention enhanced with mHealth technology that used evidence-based motivational enhancement and behavioral activation strategies to facilitate self-management of asthma and obesity symptoms in these adolescents.<br><br>This intervention provided young people with strategies and education to control/manage their health and increased their ability to self-manage their condition, medications, and associated symptoms. | *Short Assessment of Health Literacy-English (SAHL-E)*<br><br>**Asthma control** → *Asthma Control Test (ACT)*<br><br>**Beliefs related to illness** → *Asthma Belief Scale*<br><br>**Depressive symptomatology** → *PROMIS Pediatric Depressive Symptoms 8a*<br><br>**Anxiety symptomatology** → *PROMIS Pediatric Anxiety 8a*<br><br>**Pain** → *PROMIS Pediatric Pain Interference*<br><br>*Neuro Quality of Life—Pain*<br><br>**Fatigue** → *PROMIS Pediatric Fatigue 10a*<br><br>*Neuro Quality of Life—Fatigue*<br><br>**Self-efficacy related to asthma management** → *Self-Efficacy for Managing Chronic Disease – Six Items*<br><br>**Physical activity** → *Fitbit Physical Activity Tracker* | This study was based on the Pediatric Self-Management Framework. Two evidence-based behavioral change strategies (Motivational Enhancement (ME) and Behavioral Activation (BA)) were considered, specifically targeted at these young people and used within the mHealth platform (MATADORS).<br><br>MATADORS is a self-management intervention delivered via mobile phone that includes personalized content based on existing mobile health apps SAMS (medication and inhaler adherence for children with high-risk asthma) and SELFY (family-centered symptom self-management for children with sickle cell anemia).<br><br>Behavioral change strategies were used to improve motivation within a behavioral activation (BA) framework (Motivational Enhancement (ME) behavioral change strategies within a BA framework). | Online | 6 weeks | Physical wellbeing<br><br>Cognitive coping |
| Ramsey et al. | 2022 (USA) | Persistent asthma | 26 adolescents | 12 – 17 years | Randomized clinical pilot trial | The adolescents received a phased digital intervention aimed at improving adherence to inhaled controller medication in persistent moderate-to-severe asthma cases.<br><br>This intervention included the following:<br>Step 1 → After the initial four weeks, participants received daily digital reminders for their medication through the MedaCheck Habit app, and their compliance rates were calculated. Participants whose average adherence was less than 68% were eligible for the telehealth behavioral intervention.<br><br>Step 2 → This consisted of four weekly remote behavioral intervention sessions and access to feedback on compliance via the BreatheSmart app. The manualized intervention sessions included self-management strategies, discussions about individual barriers to compliance and assigning responsibility for treatment, organizational strategies to improve compliance, and guided training in adapted problem solving. | **Treatment adherence** → the Cohero electronic monitoring system (including a Bluetooth sensor cover for the inhaler) was used to objectively assess adherence<br><br>**Illness severity and control** → Composite Asthma Severity Index (CASI) & Asthma Control Test (ACT)<br><br>**Pulmonary function** → mobile spirometer (SpirobankSmart) | Not specified | Online | Step 1 → average duration of 8 weeks<br><br>Step 2 → average duration of 5 weeks | Physical wellbeing<br><br>Cognitive coping |
| Rhee et al. | 2020 (USA) | Persistent asthma | 40 adolescents | 12 – 17 years | Randomized controlled trial | The adolescents participated in the PLASMA intervention (Peer-Led Asthma Self-Management Program for Adolescents) which was a program that aimed to improve asthma self-management and medical outcomes. It consisted of four sessions that addressed different aspects related to this disease. It was developed based on the recognition of the role that program leaders play in the success of a health program aimed at adolescents. Adolescents' perception that their leaders are similar to them can improve the program's effectiveness, especially in key areas such as age and health problems. The similarities between leaders and students are essential to improving students' receptivity to the information provided. | *Fidelity checklist by observers*<br><br>*Fidelity checklist by campers*<br><br>*Bimonthly contact checklist* | Not specified | In-person | | Physical wellbeing |
| Rodríguez et al. | 2024 (USA) | Asthma | 280 adolescent–caregiver dyads | 8 – 14 years | Single-center randomized controlled trial (parallel) | The first group participated in the A2A (*Adapt 2 Asthma*) intervention which was a bilingual, family-based program designed to teach asthma knowledge, control skills, and coping skills through interactive and culturally relevant activities for children and their parents. The culturally relevant content of this intervention included facts and myths related to cultural beliefs about asthma, conducting a motivational interviewing activity (pros and cons) related to the use of controller medications and complementary and alternative medicine strategies, and leveraging family and community support to manage the child's asthma.<br><br>The second group received the APK intervention which included a study plan that had the same number of sessions as A2A and covered identical content on asthma control. The APK did not include content on coping with the disease or culturally adapted asthma content, which were the essential elements of the intervention being tested in this study. | **Asthma control** → *Asthma Control Test (ACT)*<br><br>**Coping** → *Response to Stress Questionnaire – Pediatric Asthma Version (RSQ)* (youth self-reported, parent report of child and parent self-reported versions)<br><br>**Quality of life** → *Pediatric Quality of Life Questionnaire (PedsQL)* (child version and parent report of the child version)<br><br>**Pulmonary function** (FEV1)<br><br>**School attendance, hospital admissions and emergency room visits** → *Asthma Outcomes Questionnaire (AOQ)*<br><br>**Family asthma management** → *Family Asthma Management Symptom Scale (FAMSS)*<br><br>**Mood and behavioral symptoms in children** → *Strengths and Difficulties Questionnaire (SDQ)*<br><br>**Parents' depressive symptoms** → *Center for Epidemiological Studies Depression Scale – Short Form (CES-D Short Form)* | The A2A coping content was adapted from previously tested primary and secondary coping skills interventions to prevent depression in young people (PASCET, and Act & Adapt) | In-person | A2A → seven sessions of 1 hour<br><br>APK → seven sessions | Physical wellbeing<br><br>Emotional wellbeing<br><br>Cognitive coping |

| | | | | | | | | | | | |
|---|---|---|---|---|---|---|---|---|---|---|---|
| Taheri et al. | 2022<br>(Iran) | Asthma | 72 adolescents | 10 – 15 years | Randomized controlled trial | The participants in the experimental group received an intervention consisting of a motivational interview during five weekly sessions focused on motivating adolescents and exploring and resolving ambivalence about adherence to asthma medication and disease control behaviors.<br><br>Adolescents in the control group received only usual medical care (no psychological or motivational interventions, except in special cases). | **Asthma-related self-efficacy** → *Asthma Self-efficacy Questionnaire (ASQ)*<br><br>**Treatment adherence** → *Adherence self-report form*<br><br>**Asthma control** → *Asthma control questionnaire* | Not specified | In-person | Five weekly sessions of 80-90 minutes each | Physical wellbeing<br><br>Cognitive coping |
| Tseng et al. | 2020<br>(Taiwan) | Asthma | 90 adolescents | 12 – 18 years | Randomized controlled trial (two parallel groups) | The participants received the "*Asthma Self-Management* Program (ASMP)" intervention. This program consists of three face-to-face sessions, followed by two short text messages and a telephone call. | **Asthma-related self-efficacy, outcome expectations, asthma-related prevention and management behaviors, and asthma symptom control** → *Asthma Self-Efficacy Index; Outcome Expectancy; Asthma Prevention and Management Index; Asthma Control Test (ACT)* | Bandura's self-efficacy model | In-person | Three sessions<br>(4 weeks of duration) | Physical wellbeing |
| Wang et al. | 2025<br>(China) | Asthma | 58 dyads (28 in each group) | 6-12 years | Randomized controlled pilot study (two parallel groups) | The dyads in the experimental group received an *Acceptance and Commitment Therapy-based empowerment* intervention which was offered in a hybrid group format (six to eight families). It consisted of three components: (a) ACT for parents, (b) family empowerment strategies, and (c) training in asthma education and self-management.<br><br>The parent–child dyads in the control group received standard asthma care from pediatric respiratory clinics. | **Parental stress** → *Parental Stress Scale*<br><br>**Asthma control** → *Childhood Asthma Control Test*<br><br>**Anxiety symptoms** → *Generalized Anxiety Disorder-7*<br><br>**Depressive symptoms** → *Patient Health Questionnaire-9*<br><br>**Psychological flexibility** → *PsyFlex questionnaire*<br><br>**Familiar empowerment** → *Family Empowerment Scale*<br><br>**Asthma knowledge** → *Asthma Knowledge Questionnaire*<br><br>**Asthma management abilities** → *Chinese Self-Management Scale for Asthmatic Children* | The intervention was developed in accordance with the "Medical Research Council framework for complex interventions". | Hybrid (online and in-person) | Five consecutive weekly sessions<br><br>Total duration of 9 hours | Physical wellbeing<br><br>Identity<br><br>Social and support relationships |
| Weinstein et al. | 2020<br>(USA) | Uncontrolled asthma | 223 dyads | 5-16 years | Randomized comparative study | Families assigned to the AE-C group (interventions with certified asthma educators) discussed asthma symptoms, control, triggers, technique and compliance with pharmacological treatment, asthma plans, and other issues. These families received a follow-up phone call after each session.<br><br>For families assigned to CHW (interventions using community health workers), the CHW protocol was followed, which consisted of standard asthma education topics to reduce deterioration and risk, as well as weekly behavioral change plans. Each visit aimed to cover one or more topics from the core curriculum; if any barriers were identified, self-management skills (problem solving, self-management, environmental reorganization, and social support) were introduced. The wellbeing of parents and children was also addressed at least once in most families, including stress, anxiety, depression, and referrals to behavioral problems. | **Asthma control** → *ACT and cACT*<br><br>**Parents mental health** → *Nine-item Patient Health Questionnaire; Six-item Short Form of the PTSD Checklist—Civilian Version*<br><br>**Children mental heal**th → *Children's Depression Inventory 2, Short Form; Patient-Reported Outcome Measurement Information System Depressive Symptoms Parent Proxy form; Child Report of Post-Traumatic Symptoms; Parent Report of Post-Traumatic Symptoms; modified version of Traumatic Event Screening Inventory—Child Report Form Revised*<br><br>**Family functioning** → *Chaos, Hubbub, and Order Scale (CHAOS)* | Programs based on CHWs (Community Health Workers) aim to empower families to develop problem-solving skills, improving self-efficacy and the sustainability of positive outcomes. A flexible program allows modules to be adapted to the specific needs of each family. | In-person | AE-C group → one session of 1 hour during the next month to the randomization and one session of follow-up after 6 months (these families also received follow-up through phone call after each session).<br><br>CHW group → 10 visits over 12 months. | Physical wellbeing<br><br>Cognitive coping<br><br>Social and support relationships |
